# Visual and Semiquantitative Assessment of ^123^I-Ioflupane SPECT in Probable Dementia with Lewy Bodies and Its Association with Autonomic Dysfunction: A Retrospective Study

**DOI:** 10.3390/jcm15176647

**Published:** 2026-08-28

**Authors:** Tahmina Arslan, Recep Bekiş, Mehmet Selman Ontan, Ahmet Turan Isik

**Affiliations:** 1 Department of Nuclear Medicine, Cigli Training and Research Hospital, Izmir Bakircay University, 35620 Izmir, Türkiye; 2 Department of Nuclear Medicine, Faculty of Medicine, Dokuz Eylül University, 35340 Izmir, Türkiye; recep.bekis@deu.edu.tr; 3 Department of Geriatrics, Izmir Tepecik Training and Research Hospital, 35180 Izmir, Türkiye; selmanontan@gmail.com; 4 Unit for Brain Aging and Dementia, Department of Geriatric Medicine, Faculty of Medicine, Dokuz Eylül University, 35340 Izmir, Türkiye; atisik@yahoo.com

**Keywords:** dementia with Lewy bodies, ^123^I-ioflupane SPECT, dopamine transporter imaging, DaTSCAN, DaTQUANT, semiquantitative analysis, autonomic dysfunction, dementia severity

## Abstract

**Background/Objectives**: Dementia with Lewy bodies (DLB) is a clinically heterogeneous neurodegenerative disorder characterized by cognitive, neuropsychiatric, motor, and autonomic manifestations. Reduced striatal dopamine transporter availability on ^123^I-ioflupane single-photon emission computed tomography (SPECT) is an established indicative biomarker of DLB. However, the associations among expert visual interpretation, regional semiquantitative dopamine transporter measurements, autonomic manifestations, and dementia severity remain incompletely characterized. This study aimed to evaluate the relationship between visual ^123^I-ioflupane SPECT classification and regional DaTQUANT z-scores in patients with clinically probable DLB and to explore their associations with autonomic manifestations and dementia severity. **Methods**: This retrospective, cross-sectional study included 30 patients who received a clinical diagnosis of probable DLB according to the 2017 DLB Consortium criteria before the ^123^I-ioflupane SPECT results became available. SPECT images were assessed visually by experienced nuclear medicine physicians and semiquantitatively using DaTQUANT software. Bilateral striatal, putaminal, caudate, and putamen-to-caudate ratio z-scores were analyzed in relation to visual scan classification, Clinical Dementia Rating (CDR) scores, and retrospectively ascertained autonomic manifestations, including orthostatic hypotension, delayed orthostatic hypotension, supine hypertension, postprandial hypotension, constipation, and urinary incontinence. **Results**: Scans were visually classified as supportive of nigrostriatal dopaminergic degeneration in 23 of 30 patients (76.7%) and as non-supportive in seven (23.3%). Bilateral striatal, putaminal, and caudate z-scores were significantly lower in visually supportive scans than in non-supportive scans (all *p* < 0.001), whereas putamen-to-caudate ratio z-scores did not differ significantly between the groups. None of the evaluated autonomic manifestations was significantly associated with either visual scan classification or regional DaTQUANT measurements. No regional DaTQUANT measurement was significantly associated with dementia severity. A modest positive correlation was observed between the left putamen-to-caudate ratio z-score and CDR (Spearman’s ρ = 0.373, nominal *p* = 0.050); however, given the small sample size and multiple regional comparisons, this borderline finding was considered exploratory. **Conclusions**: Regional DaTQUANT measurements were consistent with expert visual interpretation of ^123^I-ioflupane SPECT in patients with clinically probable DLB. Associations of dopaminergic imaging measurements with autonomic manifestations and dementia severity were limited. Semiquantitative analysis may complement visual interpretation, but its findings should be interpreted within the broader clinical and biomarker context. Larger prospective studies incorporating standardized autonomic testing, appropriate control groups, longitudinal follow-up, and complementary biomarkers are warranted.

## 1. Introduction

Dementia with Lewy bodies (DLB) is a progressive neurodegenerative disorder pathologically characterized by the accumulation of misfolded α-synuclein in Lewy bodies and Lewy neurites throughout cortical, subcortical, brainstem, and peripheral autonomic structures. Clinically, DLB manifests as a heterogeneous constellation of progressive cognitive decline, fluctuating cognition, recurrent well-formed visual hallucinations, rapid eye movement (REM) sleep behavior disorder, spontaneous parkinsonism, autonomic dysfunction, and other neuropsychiatric manifestations. Because many of these features overlap with those of Alzheimer’s disease, Parkinson’s disease dementia, and other neurodegenerative disorders, the clinical diagnosis of DLB can be challenging, particularly during the early stages of the disease [1].

The 2017 Fourth Consensus Report of the DLB Consortium emphasizes the integration of core clinical features and indicative biomarkers in establishing a diagnosis of probable or possible DLB [1]. Reduced dopamine transporter uptake in the basal ganglia, demonstrated by single-photon emission computed tomography (SPECT) or positron emission tomography, is recognized as one such indicative biomarker. The radiotracer ^123^I-ioflupane binds to presynaptic dopamine transporters and enables visualization of nigrostriatal dopaminergic terminal integrity. Reduced striatal dopamine transporter availability is frequently observed in DLB and may aid its differentiation from dementias in which presynaptic nigrostriatal function is relatively preserved, particularly Alzheimer’s disease [2,3].

Nevertheless, dopamine transporter imaging is not disease-specific. Reduced striatal ^123^I-ioflupane uptake may also be observed in Parkinson’s disease, Parkinson’s disease dementia, multiple system atrophy, progressive supranuclear palsy, and other neurodegenerative parkinsonian syndromes [4,5]. Conversely, patients with clinically probable DLB may occasionally exhibit preserved or near-normal dopamine transporter uptake, particularly during the early stages of the disease [2]. Therefore, ^123^I-ioflupane SPECT should primarily be interpreted as a biomarker of nigrostriatal dopaminergic degeneration rather than as a stand-alone diagnostic test for DLB. Its findings should be considered together with the clinical phenotype and other available biomarkers [1,2].

In routine clinical practice, ^123^I-ioflupane SPECT is interpreted primarily through visual assessment of the intensity, symmetry, and spatial distribution of striatal tracer uptake. Although expert visual interpretation remains the conventional approach, the assessment of subtle regional abnormalities and borderline patterns may be influenced by reader experience and image quality. Semiquantitative software tools such as DaTQUANT provide standardized regional measurements relative to an age-adjusted normative database and may complement visual interpretation. Measurements of the striatum, putamen, caudate nucleus, and putamen-to-caudate ratios may therefore provide a more objective characterization of the regional distribution of dopaminergic loss. In the present study, because the visual and semiquantitative assessments were derived from the same SPECT examinations, their comparison was intended to evaluate agreement between the two methods rather than to independently validate diagnostic accuracy.

Autonomic dysfunction is another clinically important component of the DLB phenotype. Orthostatic hypotension, supine hypertension, postprandial hypotension, constipation, urinary dysfunction, and other autonomic manifestations may occur in established DLB and may also precede the development of overt dementia [6,7,8,9]. Lewy body pathology may involve both central and peripheral autonomic pathways, including brainstem nuclei and sympathetic and parasympathetic structures [8]. It is therefore biologically plausible that nigrostriatal dopaminergic degeneration and autonomic dysfunction coexist as manifestations of widespread α-synuclein pathology. However, previous studies examining the associations among reduced dopamine transporter availability, autonomic manifestations, and cognitive impairment have yielded inconsistent results [6,10].

The interpretation of these associations is further complicated by several clinical and methodological factors. In older patients with DLB, autonomic manifestations may be influenced by cardiovascular comorbidities, hydration status, disease severity, and medication use. Dopaminergic drugs, antihypertensive agents, psychotropic medications, and other treatments may affect blood pressure regulation, gastrointestinal function, and urinary symptoms independently of the underlying neurodegenerative process. Furthermore, in retrospective studies, autonomic manifestations may be incompletely documented, particularly when standardized autonomic testing is not systematically performed. These potential confounding factors and methodological limitations should therefore be considered when evaluating associations between dopaminergic imaging findings and autonomic dysfunction.

A previous study of atypical parkinsonism suggested that perfusion SPECT may provide complementary information when interpreted alongside structural magnetic resonance imaging (MRI) [11]. In that study, the combined use of perfusion SPECT and MRI contributed to the evaluation of progressive supranuclear palsy–parkinsonism predominant (PSP-P) and multiple system atrophy–parkinsonian type (MSA-P); however, the authors also highlighted the limitations of individual imaging modalities and the potential value of a multimodal assessment. Reduced myocardial uptake on cardiac ^123^I-metaiodobenzylguanidine (MIBG) scintigraphy, which assesses postganglionic cardiac sympathetic innervation, is recognized as an indicative biomarker of DLB [1,8].

Although the role of dopamine transporter SPECT as an indicative biomarker of DLB is well established, few studies have simultaneously evaluated expert visual interpretation, automated regional semiquantitative measurements, dementia severity, and autonomic manifestations within a single clinically defined DLB cohort. A key methodological feature of the present study is that the clinical classification of probable DLB was established before the ^123^I-ioflupane SPECT results became available, and the imaging findings were not used to determine study eligibility, thereby reducing incorporation bias in participant selection.

Accordingly, the primary aim of this study was to evaluate the relationship between expert visual classification of ^123^I-ioflupane SPECT images and regional semiquantitative DaTQUANT measurements in patients with clinically probable DLB. The secondary aim was to explore the associations of regional DaTQUANT measurements with autonomic dysfunction and dementia severity, as assessed using the Clinical Dementia Rating (CDR) scale. Given the retrospective, cross-sectional, and exploratory design of the study, these analyses were intended to characterize imaging–clinical associations and agreement between the two assessment methods rather than to establish diagnostic accuracy or disease-specific quantitative cutoff values.

## 2. Materials and Methods

### 2.1. Study Design and Participants

This retrospective, cross-sectional observational study included patients evaluated at the Nuclear Medicine and Geriatrics outpatient clinics of Dokuz Eylül University Faculty of Medicine between 2023 and 2024. The study aimed to assess the relationship between visual and semiquantitative ^123^I-ioflupane SPECT findings in patients with clinically probable dementia with Lewy bodies (DLB) and to explore their associations with autonomic dysfunction and dementia severity.

Patients were eligible for inclusion if they were aged ≥50 years, fulfilled the 2017 DLB Consortium criteria for clinically probable DLB [1], underwent ^123^I-ioflupane SPECT as part of their diagnostic evaluation, and had available clinical data on autonomic dysfunction. Importantly, the clinical diagnosis of probable DLB was established before the ^123^I-ioflupane SPECT findings became available; therefore, the imaging results were not used to determine study eligibility or the initial clinical diagnosis. This approach minimized incorporation bias in participant selection.

Patients were excluded if they had a prior diagnosis of Parkinson’s disease, Parkinson’s disease dementia, Alzheimer’s disease, or any other type of dementia; technically inadequate ^123^I-ioflupane SPECT images; autonomic dysfunction attributable to a known cardiovascular disorder; or any systemic or neurological condition that could substantially affect cognitive function, including advanced renal failure, malignancy, or intracranial neoplasia.

After application of the eligibility criteria, 30 patients were included in the final analysis. Demographic, clinical, cognitive, autonomic, and imaging data were retrospectively extracted from electronic and paper medical records. As no longitudinal follow-up was performed, all clinical and imaging variables were evaluated cross-sectionally.

### 2.2. Clinical Diagnosis and Cognitive Assessment

The clinical diagnosis of probable DLB was established by geriatricians in accordance with the criteria outlined in the 2017 Fourth Consensus Report of the DLB Consortium [1]. The diagnostic assessment included clinical history, functional and cognitive decline, neurological examination findings, systemic comorbidities, available neuropsychological assessments, brain magnetic resonance imaging findings, and relevant laboratory data.

The core clinical features evaluated were fluctuating cognition, recurrent well-formed visual hallucinations, rapid eye movement (REM) sleep behavior disorder, and spontaneous parkinsonism. For this study, probable DLB was operationally defined as progressive cognitive decline accompanied by at least two core clinical features. Patients who would have met the criteria for probable DLB solely on the basis of one core clinical feature plus a positive indicative imaging biomarker were not eligible under this operational definition. Accordingly, ^123^I-ioflupane SPECT findings were not used in the clinical classification for study inclusion.

Available cognitive assessments included the Mini-Mental State Examination, clock-drawing test, verbal fluency tests, and tests of attention and executive function. Dementia severity was assessed using the global Clinical Dementia Rating (CDR) score documented in the medical records. CDR scores were categorized as 0, 0.5, 1, 2, or 3 and subsequently analyzed in relation to the visual classification of ^123^I-ioflupane SPECT images and regional DaTQUANT measurements.

Importantly, the CDR was used solely as a retrospective measure of dementia severity and was not used as a diagnostic criterion for probable DLB. A global CDR score of 0 was documented in the medical record of one patient. Review of the original clinical record confirmed that this value was not attributable to a transcription error. Despite this score, the treating geriatrician had diagnosed probable DLB based on the documented history of functional and cognitive decline and the presence of at least two core clinical features. Therefore, this patient remained eligible according to the predefined clinical inclusion criteria. CDR data were unavailable for two of the 30 patients.

### 2.3. Assessment of Autonomic Dysfunction

Autonomic dysfunction was evaluated retrospectively using clinical records, documented blood pressure measurements, and patient- or caregiver-reported information recorded at the time of clinical assessment. The autonomic manifestations assessed included orthostatic hypotension, delayed orthostatic hypotension, supine hypertension, postprandial hypotension, constipation, and urinary incontinence. Each autonomic manifestation was evaluated separately in the statistical analyses.

Orthostatic hypotension was defined as a sustained decrease in systolic blood pressure of ≥20 mmHg or in diastolic blood pressure of ≥10 mmHg within 3 min of standing. Delayed orthostatic hypotension was defined as a blood pressure decrease meeting the same thresholds more than 3 min after standing. Supine hypertension was defined as a systolic blood pressure of ≥140 mmHg or a diastolic blood pressure of ≥90 mmHg in the supine position. Postprandial hypotension was defined as a decrease in systolic blood pressure of ≥20 mmHg within 2 h after a meal.

Constipation was considered present when the clinical records documented fewer than three bowel movements per week or regular laxative use for constipation. Urinary incontinence was considered present when involuntary urinary leakage was documented in the clinical history. Autonomic data were derived from patient- or caregiver-reported information documented in the medical records and from available objective clinical measurements. For some patients, additional investigations performed as part of routine clinical assessment, including ambulatory blood pressure monitoring and electrocardiography, were available and included in the retrospective review.

Owing to the retrospective study design, a dedicated prospective autonomic testing protocol was not implemented. Tilt-table testing, the Ewing autonomic battery, and standardized autonomic symptom questionnaires, such as the Composite Autonomic Symptom Score 31 (COMPASS-31), were not systematically administered across the entire cohort. Consequently, the autonomic variables analyzed in this study should be interpreted as routinely documented clinical manifestations rather than standardized laboratory measures of autonomic function.

### 2.4. ^123^I-Ioflupane SPECT Acquisition

All patients underwent ^123^I-ioflupane SPECT imaging using an Optima NM/CT 640 SPECT/CT system (GE Healthcare). All examinations were performed at a single center using a uniform acquisition and reconstruction protocol.

Thyroid blockade was administered according to the institutional protocol at least 1 h before radiotracer injection. Subsequently, 111–185 MBq (3–5 mCi) of ^123^I-ioflupane was administered intravenously. Image acquisition was performed 3–6 h after radiotracer administration.

SPECT images were acquired using low-energy high-resolution (LEHR) collimators. The photopeak was centered at 159 keV using a ±10% energy window. A total of 120 projections were acquired over a 360° rotation using a 128 × 128 acquisition matrix, with an acquisition time of approximately 20–30 s per projection. Images were reconstructed using ordered-subsets expectation maximization (OSEM), with uniform reconstruction parameters applied to all patients. The imaging procedures followed established recommendations for presynaptic dopaminergic SPECT imaging [12].

### 2.5. Visual Image Interpretation

SPECT images were visually assessed by two experienced nuclear medicine physicians. Visual interpretation focused on the intensity, symmetry, and spatial distribution of ^123^I-ioflupane uptake in the caudate nuclei and putamina bilaterally.

Scans with preserved and relatively symmetric striatal tracer uptake were classified as visually non-supportive of nigrostriatal dopaminergic degeneration, whereas those with asymmetric or diffusely reduced striatal uptake were classified as visually supportive.

### 2.6. Semiquantitative DaTQUANT Analysis

Semiquantitative analysis was performed using DaTQUANT software, version 2.0000 (GE Medical Systems Israel, Functional Imaging, Tirat Hacarmel, Israel). The software provided measurements of the whole striatum, putamen, and caudate nucleus on each side. Specific binding ratios (SBRs) were calculated for the predefined striatal regions and compared with the software’s age-adjusted normative reference database to generate regional z-scores. Putamen-to-caudate ratios and their corresponding z-scores were also recorded.

The semiquantitative variables included in the analyses were the right and left striatal, putaminal, caudate, and putamen-to-caudate ratio z-scores.

DaTQUANT z-scores indicate the position of each software-derived regional measurement relative to the normative reference distribution. Negative z-scores represent values below the normative reference mean, whereas positive z-scores represent values above it. Accordingly, large positive z-scores were interpreted as measurements above the normative reference mean rather than as evidence of pathologically increased dopaminergic activity.

All DaTQUANT values included in the statistical analyses were extracted directly from the original semiquantitative outputs. Because the visual classifications and DaTQUANT measurements were derived from the same SPECT examinations, their comparison was interpreted as an assessment of agreement between the two methods rather than as an independent validation of diagnostic accuracy.

### 2.7. Statistical Analysis

Statistical analyses were performed using SPSS for Windows, version 15.0 (SPSS Inc., Chicago, IL, USA). Categorical variables were summarized as frequencies and percentages, whereas continuous variables were presented as means ± standard deviations and ranges.

Categorical variables were compared using the chi-square test or Fisher’s exact test, as appropriate. Continuous variables were compared between two independent groups using the independent-samples Student’s *t*-test when the assumptions for parametric testing were satisfied and the Mann–Whitney U test otherwise. Comparisons involving more than two groups were performed using one-way analysis of variance when parametric assumptions were satisfied and the Kruskal–Wallis test otherwise.

Associations between regional DaTQUANT measurements and ordinal CDR scores were evaluated using Spearman’s rank correlation analysis.

Receiver operating characteristic (ROC) curve analyses were performed to explore the ability of regional DaTQUANT z-scores to distinguish between visually supportive and visually non-supportive scans. Because the visual classifications and DaTQUANT measurements were derived from the same SPECT examinations, the resulting areas under the curve (AUCs) were not interpreted as estimates of diagnostic accuracy for DLB. Instead, they were considered exploratory measures of discrimination relative to the visual assessment.

A two-sided nominal *p*-value < 0.05 was considered statistically significant. Because multiple regional imaging variables were evaluated in a relatively small cohort without a prespecified correction for multiple comparisons, isolated borderline results were interpreted as exploratory. Specifically, the association with a nominal *p*-value of 0.050 was not considered statistically significant or confirmatory.

### 2.8. Ethical Approval

The study was conducted in accordance with the Declaration of Helsinki and was approved by the Dokuz Eylül University Non-Interventional Research Ethics Committee (file No. 9593-GOA; decision No. 2025/09-04; approval date: 12 March 2025). The requirement for informed consent was waived by the ethics committee because of the retrospective study design and the use of anonymized clinical and imaging data.

## 3. Results

### 3.1. Patient Characteristics

A total of 30 patients with clinically probable DLB were included in the study. The mean age was 75.7 ± 7.8 years (range: 59–91 years), and 16 patients (53.3%) were female. Diabetes mellitus was present in 16 patients (53.3%) and hypertension in 20 (66.7%). Ten patients (33.3%) were receiving antiparkinsonian medications, and two (6.7%) were receiving antipsychotic medications.

CDR data were available for 28 of the 30 patients. Among these patients, one (3.6%) had a recorded CDR score of 0, eight (28.6%) had a score of 0.5, 13 (46.4%) had a score of 1, and six (21.4%) had a score of 2. No patient had a recorded CDR score of 3. The patient with a CDR score of 0 was retained in the analysis because review of the original clinical record confirmed that the treating geriatrician had established the diagnosis of probable DLB based on the documented history of functional and cognitive decline and the presence of at least two core clinical features, independently of the CDR score.

Autonomic manifestations were common in the cohort. Orthostatic hypotension within 3 min of standing was documented in 10 patients (33.3%), delayed orthostatic hypotension in three (10.0%), constipation in 10 (33.3%), and urinary incontinence in 20 (66.7%). Supine hypertension was documented in 18 of 29 evaluable patients (62.1%), whereas postprandial hypotension was documented in 22 of 26 evaluable patients (84.6%) (Table 1).

### 3.2. Visual and Semiquantitative ^123^I-Ioflupane SPECT Findings

Scans were visually classified as supportive of nigrostriatal dopaminergic degeneration in 23 of 30 patients (76.7%), whereas seven patients (23.3%) had visually non-supportive scans.

Regional DaTQUANT measurements differed significantly according to visual scan classification. Patients with visually supportive scans had significantly lower bilateral striatal, putaminal, and caudate z-scores than those with visually non-supportive scans (all *p* < 0.001). In contrast, neither the right nor the left putamen-to-caudate ratio z-score differed significantly between the two groups (*p* = 0.677 and *p* = 0.625, respectively) (Table 2).

**Figure 1:**
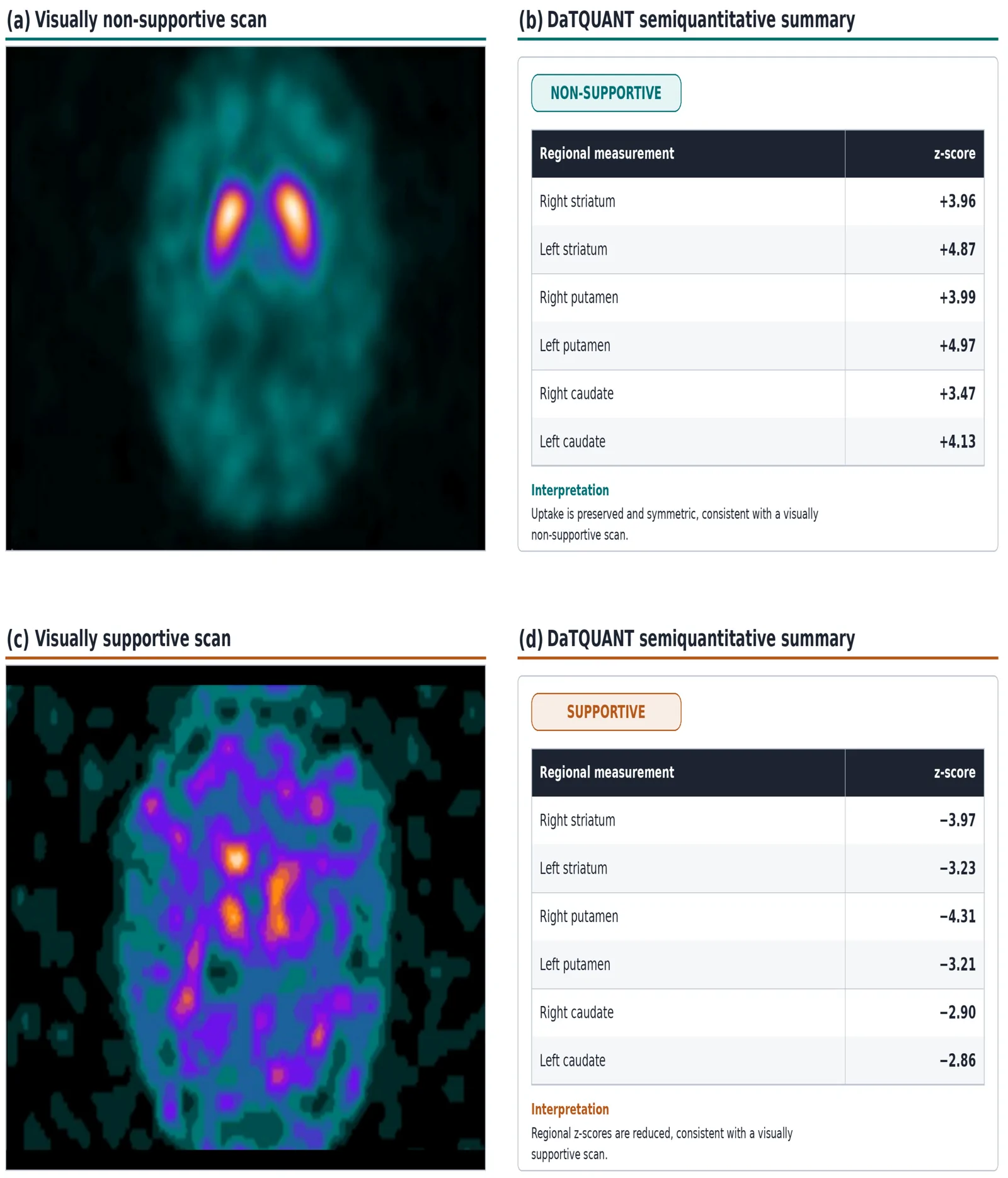
Representative visual and semiquantitative ^123^I-ioflupane SPECT findings. (**a**) A visually non-supportive scan showing preserved and relatively symmetric bilateral striatal tracer uptake. (**b**) The corresponding DaTQUANT semiquantitative output showing regional z-scores relative to the software’s age-adjusted normative reference database. (**c**) A visually supportive scan showing markedly reduced striatal tracer uptake. (**d**) The corresponding DaTQUANT semiquantitative output demonstrating lower regional z-scores relative to the normative reference mean.

The greatest numerical between-group difference was observed in the left putamen, for which the mean z-score was −1.37 ± 1.58 in the visually supportive group, compared with 2.55 ± 1.89 in the visually non-supportive group. Similar differences were observed in the right putamen and in the bilateral striatal and caudate measurements. Representative visual and semiquantitative findings are shown in Figure 1.

### 3.3. Associations with Autonomic Dysfunction and Dementia Severity

None of the evaluated autonomic manifestations differed significantly between patients with visually supportive and visually non-supportive SPECT findings. Orthostatic hypotension within 3 min of standing was present in 34.8% of patients with visually supportive scans and 28.6% of those with visually non-supportive scans (*p* = 1.000). Delayed orthostatic hypotension was observed in 13.0% and 0%, respectively (*p* = 1.000). The prevalence of supine hypertension, constipation, urinary incontinence, and postprandial hypotension likewise did not differ significantly between the two visual groups (all *p* > 0.05).

Similarly, none of the individual autonomic manifestations was significantly associated with the regional DaTQUANT z-scores. No significant associations were observed between CDR categories and any of the evaluated autonomic manifestations (all *p* > 0.05).

No regional DaTQUANT measurement was significantly correlated with dementia severity, as assessed by the CDR score. Correlation coefficients for the bilateral striatal, putaminal, and caudate z-scores were close to zero. The right putamen-to-caudate ratio z-score showed a nonsignificant positive correlation with CDR (Spearman’s ρ = 0.317, *p* = 0.100). The left putamen-to-caudate ratio z-score showed a modest positive correlation with CDR (Spearman’s ρ = 0.373, nominal *p* = 0.050); however, given the small sample size, multiple regional comparisons, and absence of a prespecified correction for multiple testing, this borderline finding was considered exploratory and was not interpreted as confirmatory.

### 3.4. Exploratory Discrimination Relative to Visual Classification

Receiver operating characteristic (ROC) curve analyses were performed to explore the ability of regional DaTQUANT z-scores to distinguish between visually supportive and visually non-supportive scans. Because the visual classifications and DaTQUANT measurements were derived from the same ^123^I-ioflupane SPECT examinations, the resulting areas under the curve (AUCs) were interpreted as measures of discrimination relative to visual assessment rather than as estimates of diagnostic accuracy for DLB.

Among the regional measurements, the left putamen z-score yielded the largest AUC (0.994; 95% CI: 0.873–1.000), followed by the left striatum (AUC = 0.969; 95% CI: 0.831–0.999), right striatum (AUC = 0.944; 95% CI: 0.794–0.995), right putamen (AUC = 0.944; 95% CI: 0.794–0.995), left caudate (AUC = 0.938; 95% CI: 0.786–0.993), and right caudate (AUC = 0.901; 95% CI: 0.736–0.979). In contrast, the right and left putamen-to-caudate ratio z-scores showed limited discrimination relative to the visual classification, with AUCs of 0.590 (*p* = 0.410) and 0.571 (*p* = 0.532), respectively.

These results should therefore be interpreted as exploratory measures of how well semiquantitative measurements distinguish between visual categories within the same imaging dataset, rather than as estimates of diagnostic performance for DLB. The exploratory ROC curves are shown in Supplementary Figure S1.

## 4. Discussion

In this retrospective study of patients with clinically probable DLB, ^123^I-ioflupane SPECT findings were visually supportive of nigrostriatal dopaminergic degeneration in 23 of 30 patients (76.7%). Bilateral striatal, putaminal, and caudate DaTQUANT z-scores were significantly lower in visually supportive scans, whereas putamen-to-caudate ratio z-scores did not differ between the visual groups. Autonomic manifestations were common in the cohort; however, none were significantly associated with either the visual classification or regional semiquantitative SPECT measurements. Similarly, no regional DaTQUANT measurement was significantly associated with dementia severity. The left putamen-to-caudate ratio z-score showed a modest positive correlation with CDR, but the nominal *p*-value of 0.050 was considered borderline and exploratory rather than confirmatory.

A principal finding of the present study was the strong relationship between expert visual classification and regional DaTQUANT measurements. Patients with visually supportive scans had markedly lower bilateral striatal, putaminal, and caudate z-scores than those with visually non-supportive scans. These findings are consistent with the established role of reduced striatal dopamine transporter availability as an indicative biomarker of DLB [1,2,3]. Semiquantitative analysis may therefore provide an objective complement to conventional visual interpretation, particularly in scans with subtle or borderline patterns. Nevertheless, because DaTQUANT measurements and visual classifications are derived from the same SPECT examination, their relationship reflects consistency between two assessment approaches rather than independent validation of diagnostic accuracy.

This distinction is particularly important when interpreting the exploratory ROC analyses. The left putamen z-score yielded the largest AUC and showed the strongest discrimination between visually supportive and visually non-supportive scans. However, these findings do not represent diagnostic accuracy for DLB because the visual classifications and semiquantitative measurements were derived from the same imaging dataset, and no independent reference standard, disease-control group, or neuropathological confirmation was available. The ROC findings should therefore be interpreted as exploratory measures of semiquantitative discrimination relative to visual assessment rather than as evidence of diagnostic accuracy or independent validation.

Seven patients who fulfilled the clinical criteria for probable DLB had visually non-supportive ^123^I-ioflupane SPECT scans. Preserved or near-normal striatal dopamine transporter uptake has previously been reported in a minority of patients with clinically diagnosed DLB, particularly during earlier disease stages or when nigrostriatal degeneration is not yet sufficiently pronounced to be detected by SPECT [2]. This apparent discordance may also reflect the clinical heterogeneity of DLB and its overlap with other neurodegenerative disorders. Consequently, a visually non-supportive ^123^I-ioflupane SPECT scan does not, by itself, exclude DLB; imaging findings should be interpreted together with the overall clinical presentation and other available biomarkers [1,2]. Furthermore, dopamine transporter imaging can demonstrate presynaptic nigrostriatal degeneration but cannot reliably distinguish DLB from Parkinson’s disease, Parkinson’s disease dementia, multiple system atrophy, or progressive supranuclear palsy [4,5].

The semiquantitative findings also require cautious interpretation. DaTQUANT z-scores are standardized against an age-adjusted normative reference database, with negative and positive values indicating measurements below and above the normative mean, respectively. In the present dataset, several regions yielded relatively large positive z-scores. These values should not be interpreted as evidence of pathologically increased dopaminergic activity; rather, they indicate that the software-derived regional binding measurements were above the normative reference mean. The present study was not designed to evaluate the technical factors that may influence extreme positive z-scores, such as reconstruction and normalization procedures, characteristics of the reference database, or region-of-interest definition. Accordingly, the absolute magnitude of these values should be interpreted cautiously. Importantly, the principal finding of this study was based on between-group differences in regional z-scores rather than on attributing biological significance to isolated highly positive values.

Autonomic dysfunction was common in the present cohort. Orthostatic hypotension, supine hypertension, postprandial hypotension, constipation, and urinary incontinence were frequently documented, consistent with the recognized involvement of central and peripheral autonomic pathways in Lewy body disease [8,9]. Autonomic manifestations may precede cognitive decline and become clinically prominent during the course of DLB [7,8]. Despite their high prevalence, none of the evaluated autonomic manifestations was significantly associated with either the visual ^123^I-ioflupane SPECT classification or regional DaTQUANT measurements.

Several factors may account for the absence of significant associations. Nigrostriatal dopaminergic degeneration and autonomic dysfunction may reflect the involvement of partially distinct neural systems despite arising within the context of widespread α-synuclein pathology. Dopamine transporter SPECT primarily assesses presynaptic nigrostriatal terminals, whereas autonomic manifestations may result from pathology involving brainstem autonomic nuclei, peripheral sympathetic and parasympathetic pathways, or postganglionic cardiac sympathetic fibers. Previous studies have likewise reported inconsistent associations among autonomic dysfunction, cognitive impairment, and dopaminergic imaging parameters in Lewy body disorders [6,10].

An additional consideration is the potential confounding effect of pharmacotherapy. Autonomic manifestations in patients with DLB may be influenced by dopaminergic medications, antihypertensive drugs, psychotropic agents, and other treatments affecting blood pressure regulation, gastrointestinal motility, or urinary function. In the present cohort, one-third of the patients were receiving antiparkinsonian medications, whereas a smaller proportion were receiving antipsychotic medications. Because medication exposure was not standardized and the retrospective data did not permit detailed adjustment for medication class, dose, timing, or treatment changes, residual pharmacological confounding cannot be excluded. This limitation should be considered when interpreting the absence of significant associations between autonomic manifestations and dopaminergic imaging findings.

The retrospective ascertainment of autonomic dysfunction represents another important methodological limitation. Although standardized clinical blood pressure definitions were applied when relevant measurements were available, autonomic manifestations were identified primarily from routine medical records, patient- or caregiver-reported information documented in those records, and routine clinical measurements. Dedicated autonomic laboratory assessments, such as tilt-table testing and the Ewing autonomic battery, and standardized symptom instruments, such as COMPASS-31, were not systematically administered across the entire cohort. Consequently, mild or intermittent autonomic abnormalities may have been underrecognized, and variability in clinical documentation may have introduced misclassification. These limitations may have reduced the ability to detect associations between autonomic manifestations and imaging measurements.

With respect to dementia severity, regional DaTQUANT measurements were generally not associated with CDR scores. This lack of association suggests that the severity of cognitive and functional impairment in DLB may not be explained solely by the extent of nigrostriatal dopaminergic degeneration. Cognitive impairment in DLB is multifactorial and may reflect cortical Lewy body pathology, cholinergic dysfunction, vascular changes, and coexisting Alzheimer’s disease–related amyloid and tau pathology [2,13]. Previous studies have similarly reported weak or absent associations between striatal dopamine transporter binding and clinical measures of cognitive or motor function [5].

Conversely, more detailed regional analyses have suggested that dopaminergic degeneration in specific nigrostriatal subregions may be associated with the severity of cognitive impairment. Lee et al. reported associations between lower regional dopamine transporter binding and greater cognitive impairment in Lewy body disease [14]. In the present study, the left putamen-to-caudate ratio z-score showed a modest positive correlation with CDR (Spearman’s ρ = 0.373, nominal *p* = 0.050). However, this finding was observed in a small cohort and emerged from multiple regional comparisons without a prespecified correction for multiple testing. Accordingly, it should be considered hypothesis-generating and should not be interpreted as evidence of a definitive association between the putamen-to-caudate ratio and dementia severity.

Other nuclear medicine techniques may provide complementary information beyond dopamine transporter imaging. Reduced myocardial uptake on cardiac ^123^I-metaiodobenzylguanidine scintigraphy, which assesses postganglionic cardiac sympathetic innervation, is recognized as an indicative biomarker of DLB [1,8]. In addition, cerebral perfusion SPECT may characterize regional cortical and subcortical functional abnormalities that are not directly captured by dopamine transporter imaging. Alster et al. suggested that ^99m^Tc-HMPAO perfusion SPECT could provide complementary information in the assessment of clinically overlapping atypical parkinsonian syndromes, particularly when interpreted alongside structural magnetic resonance imaging [11]. Although that study investigated PSP-P and MSA-P rather than DLB, it illustrates the broader principle that different nuclear medicine techniques assess distinct components of neurodegenerative disease and may therefore provide complementary rather than interchangeable information.

Future studies of DLB may therefore benefit from multimodal approaches combining presynaptic dopamine transporter imaging with cardiac MIBG scintigraphy, cerebral perfusion SPECT, and other imaging or fluid biomarkers. Amyloid and tau imaging or cerebrospinal fluid biomarkers may also help characterize coexisting Alzheimer’s disease–related pathology, which can substantially influence cognitive trajectories in DLB [2,8]. Longitudinal imaging studies may further clarify whether changes in regional dopamine transporter binding parallel clinical progression or whether dopaminergic, cognitive, and autonomic manifestations evolve relatively independently.

### 4.1. Strengths and Limitations

A principal methodological strength of this study is that the clinical classification of probable DLB was established before the ^123^I-ioflupane SPECT results became available. Consequently, the imaging findings were not used to determine study eligibility, thereby reducing incorporation bias in participant selection. A second strength is the combined evaluation of expert visual interpretation and automated regional semiquantitative measurements within the same cohort. The integrated assessment of imaging findings, autonomic manifestations, and dementia severity also enabled exploratory evaluation of associations among different clinical dimensions of DLB.

Several limitations should nevertheless be acknowledged. First, this retrospective, cross-sectional, single-center study included only 30 patients, resulting in limited statistical power and generalizability. Second, the study lacked a healthy control group, a disease control group, neuropathological confirmation, and an independent diagnostic reference standard. Consequently, it cannot establish the diagnostic sensitivity, specificity, or disease-specific quantitative cutoff values of ^123^I-ioflupane SPECT or DaTQUANT measurements.

Third, the visual classifications and semiquantitative measurements were derived from the same SPECT examinations. The exploratory ROC analyses therefore assessed the ability of semiquantitative measurements to distinguish between visual categories rather than providing independent diagnostic validation. Fourth, autonomic manifestations were ascertained retrospectively from routine clinical documentation and were not systematically evaluated using standardized autonomic laboratory tests or validated symptom scales. Incomplete documentation and underrecognition of mild or fluctuating autonomic abnormalities may therefore have introduced misclassification.

Fifth, medication exposure may have influenced the autonomic manifestations. Although medication use was recorded, detailed adjustment for medication class, dose, duration, and timing relative to the autonomic assessment was not possible. Sixth, CDR data were unavailable for two patients. Among those with available CDR data, one patient had a recorded global CDR score of 0 despite meeting the clinical criteria for probable DLB based on the documented history of functional and cognitive decline and the presence of at least two core clinical features. Review of the original record confirmed that this value was not attributable to a transcription error, highlighting the limitations of using a retrospectively obtained global CDR score as a surrogate for the clinician’s overall diagnostic assessment.

Seventh, multiple associations involving regional imaging and clinical variables were explored without a prespecified correction for multiple comparisons. Consequently, borderline nominal results, particularly the correlation between the left putamen-to-caudate ratio z-score and CDR, should be considered exploratory. Finally, some DaTQUANT measurements yielded relatively large positive z-scores. These values indicate software-derived measurements above the age-adjusted normative reference mean and should not be interpreted as evidence of pathologically increased dopaminergic activity. However, the retrospective dataset did not permit detailed evaluation of all technical factors that may have influenced their absolute magnitude. Their interpretation should therefore focus on relative between-group differences rather than on isolated absolute z-scores.

### 4.2. Future Perspectives

Future prospective studies should enroll larger, preferably multicenter cohorts that include appropriate disease control groups, particularly patients with Alzheimer’s disease, Parkinson’s disease dementia, and other neurodegenerative parkinsonian syndromes. Standardized autonomic evaluations incorporating tilt-table testing, validated autonomic test batteries, and symptom instruments such as COMPASS-31 would provide more reliable phenotyping and could clarify whether specific autonomic domains are associated with regional nigrostriatal dopaminergic degeneration.

Longitudinal follow-up is also needed to determine whether patients with clinically probable DLB and initially visually non-supportive dopamine transporter scans subsequently develop detectable nigrostriatal deficits. Serial imaging could help clarify the temporal relationships among dopaminergic degeneration, cognitive decline, and autonomic manifestations. Future studies should also systematically capture medication exposure and adjust for medications that may affect autonomic function.

Finally, multimodal biomarker strategies may be particularly valuable. Combining dopamine transporter SPECT with cardiac MIBG scintigraphy, cerebral perfusion SPECT, structural MRI, amyloid or tau imaging, and cerebrospinal fluid biomarkers could provide a more comprehensive assessment of nigrostriatal dopaminergic degeneration, cortical dysfunction, autonomic involvement, and coexisting Alzheimer’s disease–related pathology in DLB. Such approaches may ultimately improve biological characterization and clinical stratification beyond what can be achieved using any single imaging modality.

## 5. Conclusions

Visual ^123^I-ioflupane SPECT classifications were consistent with regional DaTQUANT measurements in patients with clinically probable DLB. Bilateral striatal, putaminal, and caudate z-scores were significantly lower in visually supportive scans, whereas putamen-to-caudate ratio z-scores did not differ significantly between the visual categories. No significant associations were identified between dopaminergic imaging measurements and the evaluated autonomic manifestations, and regional DaTQUANT measurements were generally not associated with CDR-defined dementia severity.

Semiquantitative DaTQUANT analysis may complement expert visual interpretation of ^123^I-ioflupane SPECT; however, the findings should be interpreted within the broader clinical and biomarker context. The borderline correlation between the left putamen-to-caudate ratio z-score and CDR should be regarded as exploratory rather than confirmatory. Larger prospective multicenter studies incorporating appropriate disease control groups, standardized autonomic testing, longitudinal follow-up, and complementary biomarkers are needed to confirm these observations and clarify the relationships among nigrostriatal dopaminergic degeneration, autonomic dysfunction, and cognitive progression in DLB.

## Figures and Tables

**Table 1:** Demographic and clinical characteristics of the study population.

Characteristic	Value
Age, years	75.7 ± 7.8
Age range, years	59–91
Sex, *n* (%)	
Female	16 (53.3)
Male	14 (46.7)
Diabetes mellitus, *n* (%)	16 (53.3)
Hypertension, *n* (%)	20 (66.7)
Antiparkinsonian medication use, *n* (%)	10 (33.3)
Antipsychotic medication use, *n* (%)	2 (6.7)
CDR score, *n*/*N* (%)	
0	1/28 (3.6)
0.5	8/28 (28.6)
1	13/28 (46.4)
2	6/28 (21.4)
3	0/28 (0.0)

Note: Data are presented as mean ± standard deviation or *n* (%), unless otherwise indicated. CDR data were available for 28 patients. The patient with a recorded CDR score of 0 fulfilled the clinical criteria for probable DLB based on the treating geriatrician’s overall assessment, including the documented history of functional and cognitive decline and the presence of at least two core clinical features. CDR was not used as a diagnostic criterion for study inclusion. CDR, Clinical Dementia Rating.

**Table 2:** Regional DaTQUANT z-scores according to visual ^123^I-ioflupane SPECT classification.

DaTQUANT Parameter	Visually Supportive (*n* = 23)	Visually Non-Supportive (*n* = 7)	*p*-Value
Right striatum z-score	−1.63 ± 1.73	1.99 ± 1.92	<0.001
Left striatum z-score	−1.29 ± 1.64	2.65 ± 2.02	<0.001
Right putamen z-score	−1.71 ± 1.78	1.95 ± 1.71	<0.001
Left putamen z-score	−1.37 ± 1.58	2.55 ± 1.89	<0.001
Right caudate z-score	−1.30 ± 1.65	1.88 ± 2.26	<0.001
Left caudate z-score	−0.92 ± 1.58	2.51 ± 2.12	<0.001
Right putamen-to-caudate ratio z-score	−0.54 ± 2.58	−0.10 ± 1.70	0.677
Left putamen-to-caudate ratio z-score	−0.58 ± 1.91	−0.20 ± 1.27	0.625

Note: Data are presented as mean ± standard deviation. DaTQUANT z-scores represent the number of standard deviations by which a regional measurement differs from the mean of the software’s age-adjusted normative reference distribution. Lower z-scores indicate lower regional dopamine transporter binding relative to the normative mean. Positive z-scores indicate measurements above the normative mean and should not be interpreted as evidence of pathologically increased dopaminergic activity. A two-sided *p*-value < 0.05 was considered statistically significant.

## Data Availability

The data presented in this study are available from the corresponding author upon reasonable request. The data are not publicly available because they contain clinical information and are subject to privacy and ethical restrictions.

## Supplementary Materials

The following supporting information can be downloaded at https://www.mdpi.com/article/10.3390/jcm15176647/s1, Figure S1. Exploratory receiver operating characteristic curves illustrating discrimination between visually supportive and visually non-supportive ^123I-ioflupane SPECT categories using regional DaTQUANT z-scores. Because visual interpretation and semiquantitative measurements were derived from the same SPECT examinations, these analyses represent inter-method discrimination and should not be interpreted as estimates of diagnostic accuracy for DLB.

## Abbreviations

The following abbreviations are used in this manuscript:

AUC, area under the curve; CDR, Clinical Dementia Rating; CI, confidence interval; COMPASS-31, Composite Autonomic Symptom Score 31; DaT, dopamine transporter; DLB, dementia with Lewy bodies; HMPAO, hexamethylpropyleneamine oxime; LEHR, low-energy high-resolution; MIBG, metaiodobenzylguanidine; MMSE, Mini-Mental State Examination; MRI, magnetic resonance imaging; MSA-P, multiple system atrophy–parkinsonian type; OSEM, ordered-subsets expectation maximization; PET, positron emission tomography; PSP-P, progressive supranuclear palsy–parkinsonism predominant; REM, rapid eye movement; ROC, receiver operating characteristic; SBR, specific binding ratio; SPECT, single-photon emission computed tomography.
