# Supplementary material for: Visual and Semiquantitative Assessment of 123I-Ioflupane SPECT in Probable Dementia with Lewy Bodies and Its Association with Autonomic Dysfunction: A Retrospective Study
Source: J Clin Med. 2026 Aug 28;15(17):6647. doi: 10.3390/jcm15176647 (PMC13566361; doi:10.3390/jcm15176647)

Supplementary Materials

Figure S1. Exploratory receiver operating characteristic curves illustrating discrimination between visually supportive and visually non-supportive ^123I-ioflupane SPECT categories using regional DaTQUANT z-scores. Because visual interpretation and semiquantitative measurements were derived from the same SPECT examinations, these analyses represent inter-method discrimination and should not be interpreted as estimates of diagnostic accuracy for DLB.

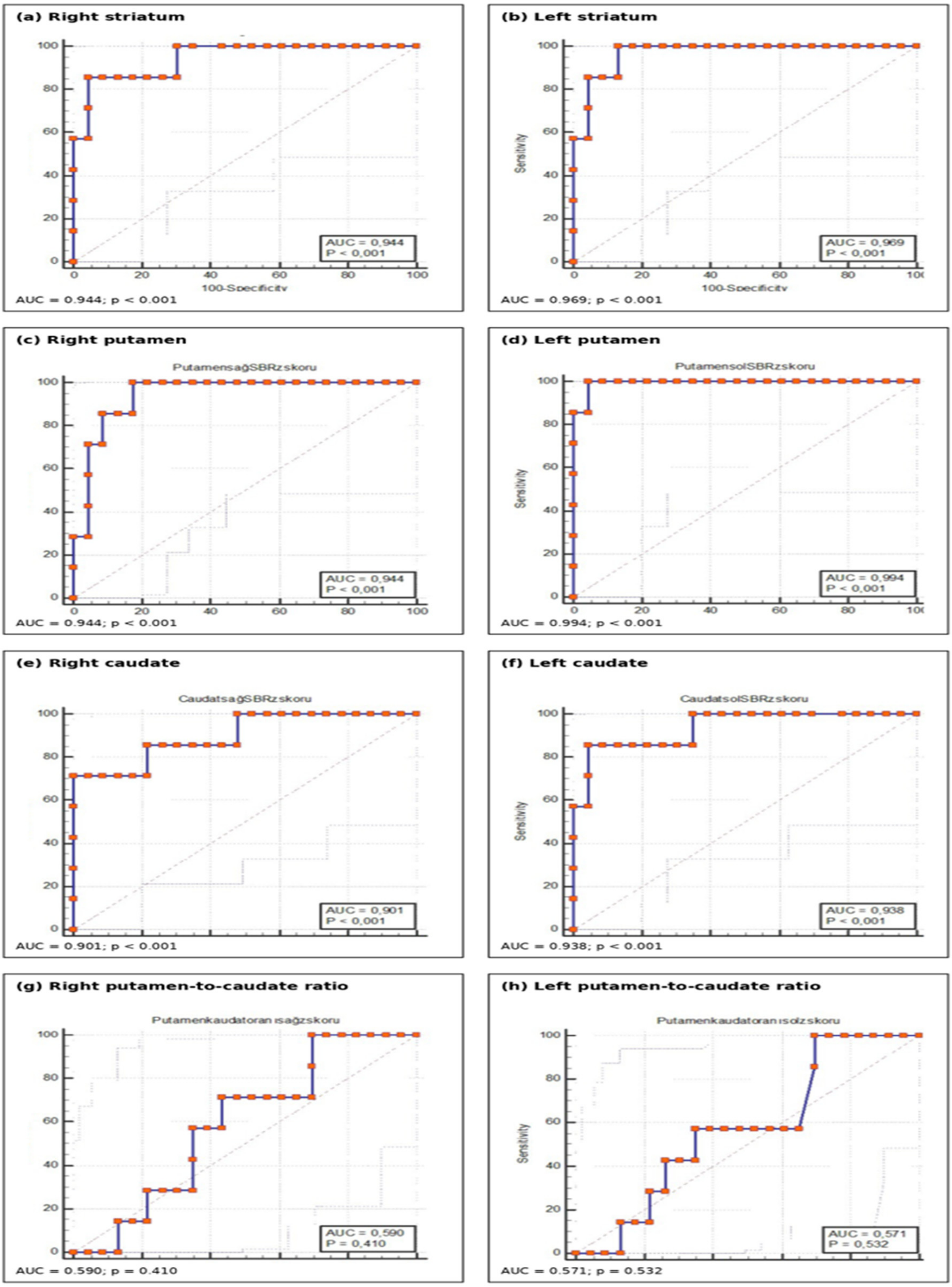

Supplement: Supplementary file 1 [file jcm-15-06647-s001.zip › jcm-4507228-supplementary.pdf]
